# Demographic and regional disparities in cancer cachexia-related mortality in the USA from 1999 to 2020 – A retrospective cross-sectional study

**DOI:** 10.1016/j.fhj.2025.100493

**Published:** 2025-12-10

**Authors:** Zahra Quettawala Mufaddal, Rafay Khan, Omer Mustafa Siddiqui, Shanza Malik, Alina Baig, Mohammad Arham Siddiq, Syed Husain Farhan, Roha Saeed Memon, Ishaque Hameed, Christopher J. Haas, Anita Tammara

**Affiliations:** a Department of Medicine, Dow University of Health Science, Karachi, Pakistan; b Department of Pathology, Sullivan Nicolaides, Springfield Lakes, Queensland, Australia; c Department of Medicine, Jinnah Sindh Medical University, Karachi, Pakistan; d Department of Medicine, Jacobi Medical Center-NYCHHC/Albert Einstein College of Medicine, Bronx, New York, USA; e Department of Medicine, Medstar Health, Baltimore, Maryland, USA

**Keywords:** Cancer outcomes, Cachexia, Trends in mortality, Racial disparities, Public health, Future health policies

## Abstract

**Background:**

Nearly 2 million new cancer cases and over half a million cancer deaths in the USA were reported in 2024. Still there remains a paucity of data on cancer-cachexia-related mortality.

**Methods:**

The Centers for Disease Control and Prevention Wide-Ranging Online Data for Epidemiologic Research (CDC WONDER) database was utilised to identify cases where both cachexia and cancer were listed as either contributory or underlying causes of death between 1999 and 2020.

**Results:**

Between 1999 and 2020, there were 64,106 deaths related to cancer and cachexia among adults aged ≥55 years. Overall, a declining trend has been identified from 1999 to 2019. Black patients had 1.4 times higher mortality than White patients. Rural dwellers had higher mortality than urban adults. Lastly, states with age-adjusted mortality rates (AAMR) in the 90th percentile (California, South Carolina, Utah, Georgia, Alaska and New Hampshire) displayed approximately five-fold higher AAMR than states in the bottom 10th percentile.

**Conclusion:**

Tailored public health strategies for healthcare access and risk factor management are needed to address these disparities.

## Highlights

• Based on the CDC WONDER database, an overall declining trend has been identified in cancer and cachexia-related mortality from 1999 to 2019.
• Age-adjusted mortality rates were notably higher in men, Black adults and rural populations.
• States in the top 90th percentile had approximately five times higher mortality rates than states in the lower 10th percentile.
• This study highlights the importance of public health strategies tailored to improve healthcare access and utilisation in population with higher mortality rates.

## Introduction

The National Center for Health Statistics projected almost 2 million new cancer cases and over half a million cancer deaths in the USA in 2024.^1^ Almost half of the cancer cases show a syndrome of cachexia characterised by anorexia and loss of adipose tissues and skeletal muscle mass.^2^ In terms of diagnostic criteria, cachexia is defined as weight loss greater than 5%, weight loss greater than 2% in individuals already showing depletion according to current body weight and height (body mass index [BMI] <20 kg/m^2^), or loss of skeletal muscle mass (sarcopenia).^3^ Cachexia, although recognised, is rarely assessed or actively managed, leading to increased morbidity and mortality. It is associated with reduced physical function, reduced tolerance to anticancer therapy, and reduced survival.^4^ In one study, a reduced median survival of 13.6 months was observed in patients with overt weight loss as compared to 28.2 months in patients without weight loss.^5^ Thus, identifying at-risk populations among patients with cancer and cachexia is crucial for timely intervention. Notably, studies have shown that the majority (75%) of individuals diagnosed with cancer cachexia were aged 56 and above.^6^ In addition, according to SEER (Surveillance, Epidemiology, and End Results Program), over 80% of cancer diagnoses, and almost all (91.6%) of cancer-related deaths occur after age 55, with the median age at death ∼73 years.^7^ Therefore, we aim to provide a comprehensive analysis of mortality trends from 1999 to 2020 among older patients (≥55 years) with cancer cachexia, with a particular focus on identifying age, racial and demographic subgroups disproportionately affected by cancer-cachexia-related mortality. This is intended to facilitate focused clinical approaches and evidence-based policy interventions to mitigate the effects such disparities.

## Methods

The Centers for Disease Control and Prevention Wide-Ranging Online Data for Epidemiologic Research (CDC WONDER) database was utilised to access data on cancer and cachexia-related mortality in the USA from 1999 to 2020.^8^ The Multiple Cause-of-Death (MCOD) public use death certificates were used to identify cases where both cachexia and cancer were listed as either contributory or underlying causes of death, on death certificates. This database has previously been utilised to analyse mortality patterns related to cancer.^9^ Cachexia was identified using the International Classification of Diseases 10th Revision (ICD-10) code R64, while cancer was identified using ICD-10 code C00-D48. We specifically focused on individuals ≥55 years because this age group accounts for >80% of cancer incidence and >90% of cancer-related mortality in the USA. This study was not subject to local institutional review board approval as it utilised deidentified government-issued publicly available data and adhered to the STROBE (Strengthening the Reporting of Observational Studies in Epidemiology) guidelines for reporting (Supplementary Table 1). We also studied cachexia-related mortality by cancer subtypes: gastrointestinal (GI) cancer (C15–C26), lung cancer (C30–C39), genitourinary (GU) cancer (C51–C58, C60–C68), prostate cancer (C61, D07.5, D29.1, D40.0), breast cancer (C50, D05), and brain cancer (C71, C72, D33, D43).

### Data abstraction

Data on primary outcomes such as overall cachexia and cancer-related deaths, population size, year, demographics, urban–rural classification and states were extracted. In addition, data on secondary outcome (location of death and cancer subtypes) were also extracted. The location of death was categorised into three main groups: medical facilities, long-term residences and other. Medical facilities comprised inpatient facilities, outpatient or ER facilities, cases of dead-on-arrival and those with unknown status in medical facilities. Long-term residences included decedents’ homes, hospice facilities and nursing homes / long-term care facilities. Demographic information (sex, race/ethnicity and age) and regional details (urban–rural classification and state) were extracted for the period spanning 1999–2020. To assess the population by urban–rural classification, the National Center for Health Statistics Urban–Rural Classification Scheme was employed, dividing counties into metropolitan (large central metropolitan, large fringe metropolitan, medium metropolitan and small metropolitan) and non-metropolitan (micropolitan and non-core) categories following the 2013 US census classification.^10^ Race and ethnicity were classified into White, Black or African American (AA), Hispanic or Latino, American Indian (AI) or Alaskan Native (AN), and Asian or Pacific Islander (PI), based on data reported on death certificates, which has been used in previous analyses of the WONDER database. The regions were classified into Northeast, Midwest, South, and West based on Census Bureau definitions.

### Statistical analysis

To analyse national trends in cachexia and cancer-related mortality, we calculated the age-adjusted mortality rates (AAMRs) per 1,000,000 population from 1999 to 2020. AAMRs were stratified by gender, race, state and metropolitan/non-metropolitan status, along with 95% confidence intervals (CIs). AAMRs were calculated by standardising cachexia and cancer-related deaths to the year 2000 US standard population as previously described.^11^

To determine the national annual trends in cachexia and cancer-related mortality, the Joinpoint Regression Program (Joinpoint V 5.2.0, National Cancer Institute) was used to identify the annual percent change (APC) with a 95% CI in AAMRs from 1999 to 2020.^12^ By fitting log-linear regression models to the data, this method identifies variations in AAMR over time, indicating increasing or decreasing trends in cachexia and cancer-related mortality. For heteroscedastic/correlated error options, the standard error provided in the dataset was applied. We allowed a maximum of four joinpoints, and the optimal number of joinpoints was selected using the data-driven Bayesian Information Criterion (weighted BIC). Joinpoints were restricted by grid search rules requiring at least two observations from a joinpoint to either end of the series, at least two observations between successive joinpoints, and testing only observed years as potential joinpoint locations (no interpolation between years). APCs and 95% CI for AAMRs were computed for the identified line segments connecting joinpoints. We used two-tailed t-testing to determine if the slope of APC describing the change in mortality was significantly different from zero. Statistical significance was set at *P* < 0.05.

## Results

### Overall cancer and cachexia-related deaths

Between 1999 and 2020, there were 64,106 deaths related to cancer and cachexia among adults aged ≥55 years. Data on the location of death were available for 63,453 patients. Over two-thirds (71.3%) of these deaths occurred in long-term residences of patients; 37.5% occurred in decedents’ homes (*n* = 23,884), 28.9% in nursing homes or long-term care facilities (*n* = 18,324), and 4.9% in hospice facilities (*n* = 3,034). The remaining deaths were distributed between medical facilities (23.4%), comprising inpatient units (21.9%, *n* = 13,921) and outpatient/ER settings (1.4%, *n* = 908). Of all deaths, 5.3% occurred in other locations (n = 3,382) (Supplementary Table 2). Of the total deaths attributed to cancer and cachexia, 84.8% occurred in White populations (*n* = 53,749), while 12.6% occurred in Black/African American patients (*n* = 8,102). The number of deaths was similar among men (52.9%) and women (47.1%) (Supplementary Table 3).

**Fig. 1:**
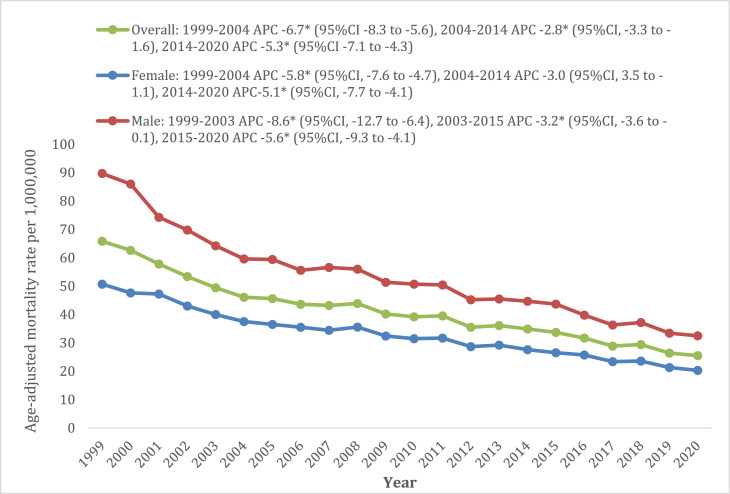
Cancer and cachexia related Age-adjusted mortality rates in the overall population aged ≥55 years old and stratified by gender from 1999 to 2020.

The overall cancer and cachexia-related mortality approximately halved over the 2 decades (1999 AAMR: 65.8, 95% CI: 63.7–67.8; 2020 AAMR: 25.5, 95% CI: 24.5–26.6). This decline was characterised by three distinct periods of change. From 1999 to 2004, mortality rates decreased significantly with an APC of −6.7% (95% CI: −8.3 to −5.6); between 2004 and 2014, the decline slowed, with an APC of −2.8% (95% CI: −3.3 to −1.6); and from 2014 to 2020, the more steep decline in mortality was apparent, with an APC of −5.3% (95% CI: −7.1 to −4.3) (Fig. 1).

### Cancer and cachexia-related AAMR stratified by gender

Throughout the study period, men had a consistently higher mortality rate than women, with an overall AAMR 1.6 times that of women (male AAMR: 51.3, 95% CI: 50.7–51.8; female AAMR: 32.3; 95% CI: 31.9–32.6). Over the 21-year period, there was a consistent decline in AAMR in men, whereas AAMR in women decreased from 1999 to 2004 (APC: −5.8%, 95% CI: −7.6 to −4.7), followed by a period of stability until 2014. Thereafter, a rapid decline in mortality was noted from 2015 to 2020 (APC: −5.6%, 95% CI: −7.7 to −4.1) (Supplementary Tables 4 and 5, Fig. 1).

### Cancer and cachexia-related AAMR stratified by race

**Fig. 2:**
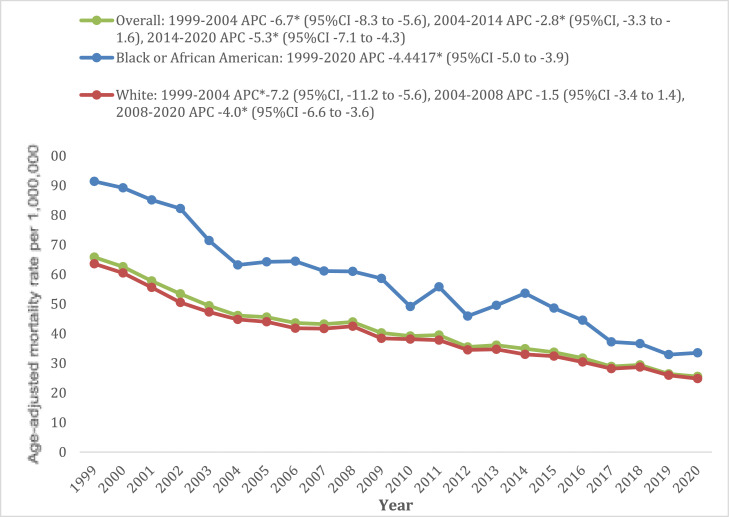
Cancer and cachexia-related age-adjusted mortality rates in the overall population aged ≥55 years old and stratified by race from 1999 to 2020.

Black/African American populations had AAMR almost 1.4 times higher than White populations. (Black/African American AAMR: 55, 95% CI: 53.7–56.2; White AAMR: 38.8, 95% CI: 38.4–39;). Black/African American populations experienced a consistent decline in mortality (APC: −4.4%, 95% CI: −5.0 to −3.9), whereas White populations experienced a steady decline from 1999 to 2004 (APC: −7.2%, 95% CI: −11.2 to −5.6), followed by a plateauing of AAMRs during 2004–2008, and subsequently, a continued decrease from 2008 to 2020 (APC: −4.0%, 95% CI: −6.6 to −3.6) (Supplementary Tables 4 and 5, Fig. 2).

### Cancer and cachexia-related AAMR stratified by region

**Fig. 3:**
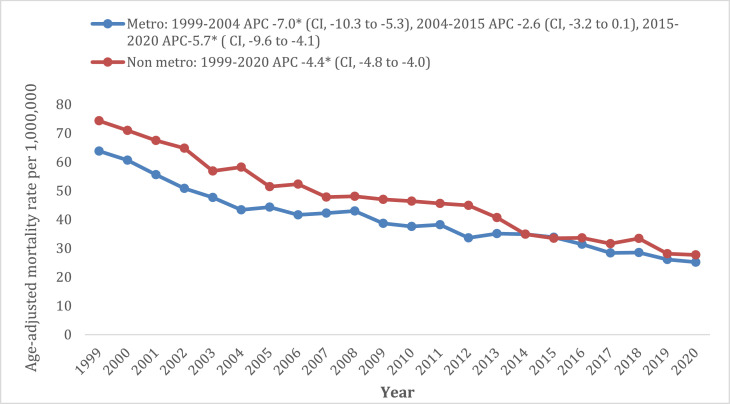
Cancer and cachexia-related age-adjusted mortality rates in population aged ≥55 years old stratified by urbanisation from 1999 to 2020.

Overall, patients in non-metropolitan locations consistently had higher AAMRs than those in metropolitan locations throughout the study period. Mortality rate in non-metropolitan areas declined with an APC of −4.4% (95% CI: −4.8 to −4.0), whereas patients in metropolitan areas had an initial decline in AAMR from 1999 to 2004 (APC: −7.0%, 95% CI: −10.3 to −5.3) after which no significant change was observed from 2004 to 2015, followed by a steady decline from 2015 to 2020 (APC: −5.7%, 95% CI: −9.6 to −4) (Supplementary Tables 5 and 6a, Fig. 3).

**Fig. 4:**
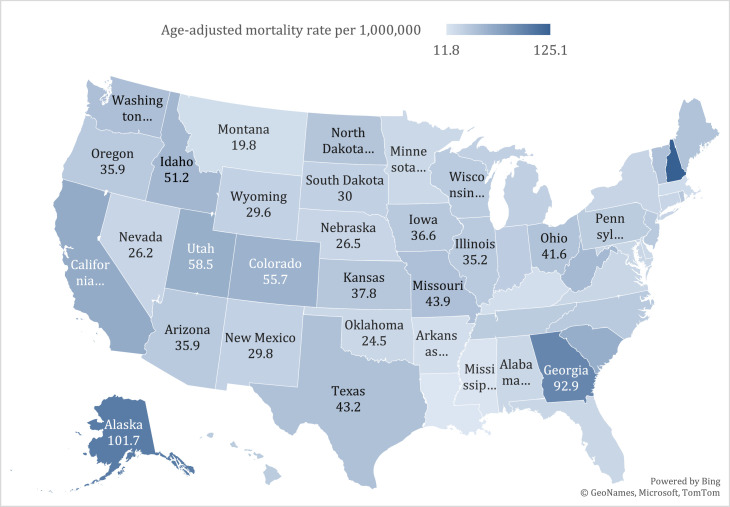
Cancer and cachexia-related age-adjusted mortality rates in population aged ≥55 years old stratified by state from 1999 to 2020.

States in the top decile (California, South Carolina, Utah, Georgia, Alaska and New Hampshire), had approximately five times higher mortality rates than states in the bottom 10th percentile (namely Louisiana, Mississippi, Arkansas, Kentucky, Massachusetts, Montana) (Supplementary Table 6b, Fig. 4).

### Cancer and cachexia-related mortality stratified by cancer subtypes

**Fig. 5:**
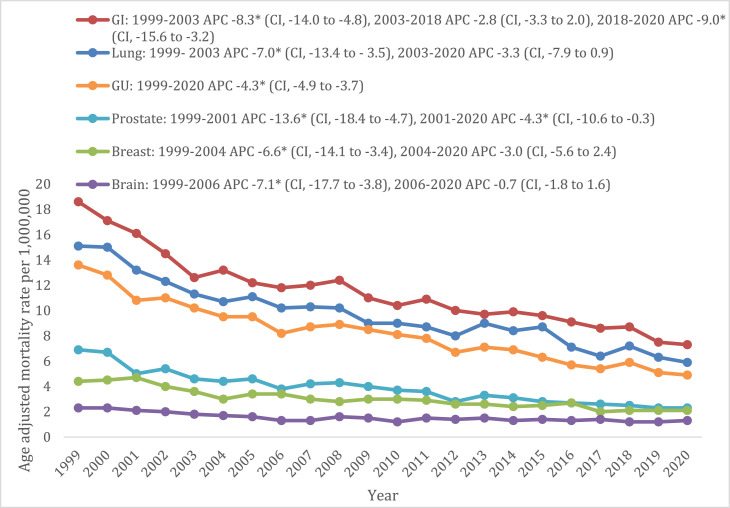
Cancer and cachexia-related age-adjusted mortality rates in the population aged ≥55 years old stratified by cancer subtypes from 1999 to 2020.

**Fig. 6:**
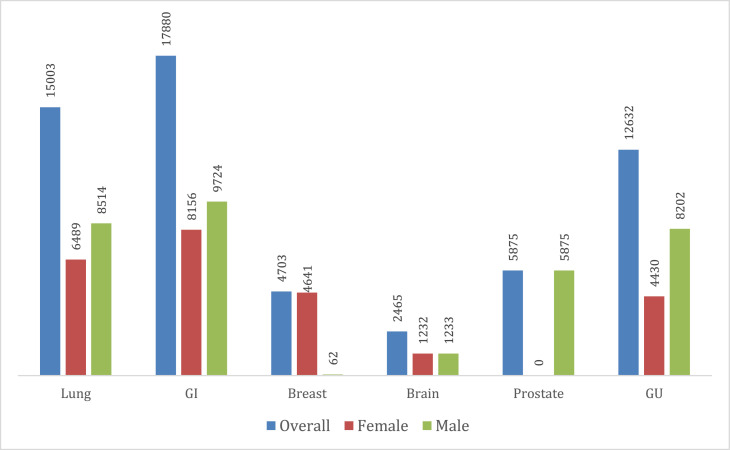
Cancer and cachexia-related total deaths in the population aged ≥55 years old stratified by cancer subtypes from 1999 to 2020.

**Fig. 7:**
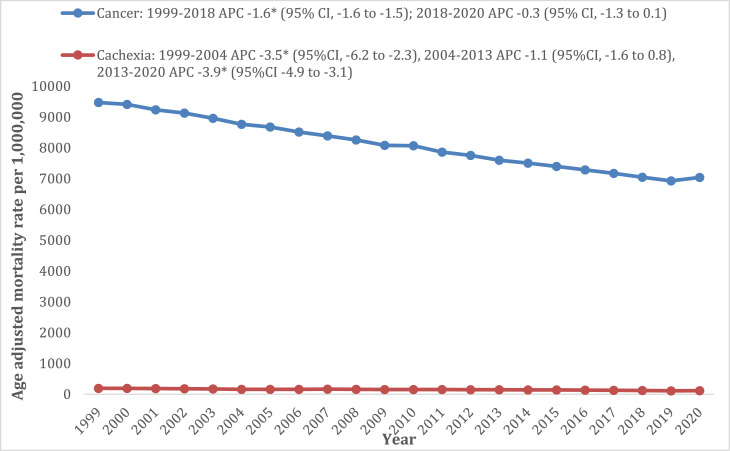
Age-adjusted mortality rate in the overall population aged ≥55 years, stratified by cancer and cachexia-related death separately.

Throughout the study period, there was a steady decline in AAMRs. Mortality rates were highest due to cachexia and GI cancers (11.1, 95% CI: 11–11.3), followed by cachexia and lung cancers (9.3; 95% CI: 9.2–9.5), cachexia and GU cancers (7.9; 95% CI: 7.8–8.1), cachexia and prostate cancers (3.7; 95% CI: 3.6–3.8), cachexia and breast cancers (2.9; 95% CI: 2.9–3) and finally, cachexia and brain cancers (1.5, 95% CI: 1.4–1.5). Notably, AAMR from GI cancers were 7.4 times that of brain cancers (Supplementary Table 7). Mortality rates due to cachexia and GU cancer and cachexia and prostate cancer declined throughout the 2 decades, whereas those due to cachexia and lung, breast and brain cancers have not decreased in the last decade after an initial steady decline (Fig. 5). Furthermore, mortality rates in men were higher for almost all cancer subtypes, except for breast cancer, in which women had a higher mortality, and brain cancer, in which mortality rates were equal (Fig. 6, Fig. 7).

## Discussion

In this 20-year analysis of cancer-related cachexia mortality data from CDC WONDER, we report several key findings (see central illustration). First, the AAMR among adults aged ≥55 years approximately halved over the last 2 decades. Second, the overall AAMR was higher among men, approximately 1.6 times compared to their female counterparts. Third, Black Americans had a consistently higher mortality than Whites. Lastly, significant regional variability existed, with states in the 90th percentile (California, South Carolina, Utah, Georgia, Alaska and New Hampshire) demonstrating a five-fold higher mortality rate compared with states in the 10th percentile (Louisiana, Mississippi, Arkansas, Kentucky, Massachusetts, Montana).

**Central:**
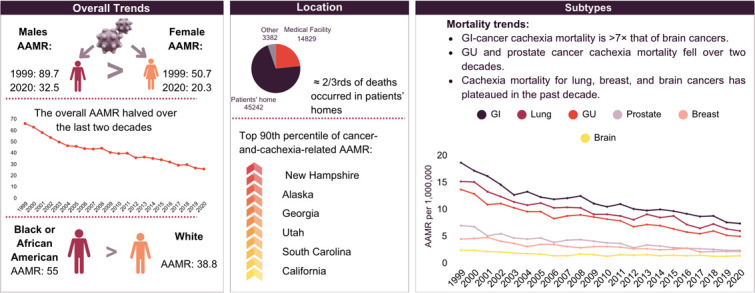
illustration.

The American Cancer Society indicates that after an increasing trend in cancer-related mortality throughout the 20th century, since 1991, cancer-related mortality has been decreasing with an overall reduction of almost 31%. This decreasing trend is similar to the results seen in this study. These trends are attributed mainly to increased smoking cessation and improvements in early detection and treatment of cancers.^13^ Our study demonstrates a higher cachexia-related mortality among cancer patients in the male population. Previous studies have demonstrated that male cancer patients have a higher incidence of cachexia with greater muscle loss and worse outcomes than their female counterparts. Various mechanisms have been theorised, including sex differences in muscle fibre type and function, mitochondrial metabolism, global gene expression and signalling pathways, and regulatory mechanisms at the levels of sex chromosomes versus sex hormones. However, further research is required to fully understand the role of genders in susceptibility leading to muscle wasting and cachexia.^14^

With respect to racial disparities, our study demonstrated a 1.4-fold higher mortality among Black individuals compared with White individuals. Previous studies have demonstrated a similar outcome with respect to GI and pancreatic cancers.^15^ This difference could be partly explained due to the social determinants affecting Black Americans. Prior studies have shown that Blacks are less likely to have routine follow-up with primary care providers and hence in part, plays a role in delayed detection of cancer.^16^

Some cancer subtypes have been shown to be consistently associated with a higher risk of cancer-related malnutrition and cachexia. This is mainly due to cancer-induced activation of inflammatory pathways.^17^ Our study demonstrated that GI and lung cancers had the highest cancer-cachexia related mortality among the cancer subtypes, a finding that aligns with the existing literature.^18^ Gastroesophageal and pancreatic cancers can cause structural/obstructive changes, anorexia and metabolic changes. Additionally, patients with pancreatic cancer may also have malabsorption due to pancreatic insufficiency, thus causing severe weight loss in these two subtypes. Furthermore, the treatment itself causes major adverse effects leading to weight loss and cachexia due to dysphagia caused by radiation therapy, odynophagia due to chemotherapy, or decreased oral intake related to anorexia.^19^ In regard to lung cancer, it has been observed that a weight loss ≥2% has been associated with poor overall and progression-free survival.^20^

Mortality rates due to cachexia among lung, breast and brain cancers remained stable in the past decade after an initial decline. A recent study published by Tan *et al* demonstrated similar outcomes among malignant brain tumours. This could be explained by multiple factors. First, the observed trends in mortality could be explained by the development of improved diagnostic tests that may have missed earlier cases in the previous years. Second, glioblastoma is the most common brain tumour among adults aged 55 and older and exhibits the lowest survival rate due to its higher disease burden.^21^ This, combined with the demographic shift towards an increasingly aging population, may contribute to the increased mortality trend seen in our study. In the population with breast cancer, no significant decline in mortality could be attributed to no major changes in the adjuvant standard of care (for adjuvant chemotherapy or hormone therapy) have taken place since 2010.^22^^,^^23^

A small retrospective study indicated that mean survival time was effectively longer (52 vs 23 days) in patients with cachexia with terminal cancer when appropriate nutritional intervention was initiated and therefore shows the significance of early recognition and treatment initiation for cancer-related cachexia.^24^ Transthyretin is a protein which has a rapid turnover of 2 days as compared to albumin (20 days) and is a well-known nutritional marker. Previously low TTR has been seen in patients with poor prognosis in cancer patients in palliative settings.^25^ It has also been seen to rapidly increase in 4–8 days in malnourished children who were given adequate nutrition.^26^ Future studies should evaluate transthyretin and other biomarkers as tools for nutritional assessment, prognostication, and monitoring of response to nutritional rehabilitation in cancer cachexia.

This study further emphasises the importance of cancer cachexia as also mentioned in the American Society of Clinical Oncology (ASCO) guidelines, which note that changes in body composition are associated with treatment toxicity, quality of life and survival. Such outcomes have initiated further research in developing biomarkers and research in therapy, including effects of already easily available medications such as mirtazapine, olanzapine and anamorelin, which show promise.^27^

Our study has several limitations. First, as the CDC WONDER extracts information from death certificates, using ICD codes to classify medical conditions carries a risk of inaccurate or missed diagnosis of cancer-related cachexia as a cause of death. This may lead to incorrect categorisation, and one study demonstrated approximately 30% of incorrect classifications in the underlying cause of death.^28^ Second, racial or ethnic categories can be incorrectly identified, and cause of death misclassified based on race, such as homicide victims being more commonly recorded as Black adults.^29^ Third, our study focused on an older population, with middle-aged and young adults excluded from the analysis, which could alter the population-level results. In addition, we focused on Black and White adults as data regarding Hispanic or Latino, American Indian (AI) or Alaskan Native (AN), and Asian or Pacific Islander (PI) were either suppressed or unreliable. Fourth, information on cancer stage and treatment status was not available. Fifth, data available on the CDC WONDER database do not include details on social determinants of health that impact access to end-of-life care. These include patient diagnoses/comorbidities, socioeconomic status, insurance coverage, prognosis and preferences, along with factors such as the degree of support from caregivers and the rate of functional decline.

## Conclusion

To summarise, cancer and cachexia-related mortality trends have steadily declined from 1999 to 2020. Significant disparities have been noted, with men faring worse than women and Black adults displaying worse outcomes than White adults. Furthermore, rural dwellers have poorer outcomes and require better access to healthcare to address such disparities. Targeted changes in policies should be made to address the underlying causes of such disparities, such as increasing the rate of healthcare utilisation among men, diminishing financial and social barriers to equitable healthcare among all ethnicities/races, and increasing the number of specialised centres in rural areas. However, these patterns should be interpreted as correlations rather than causal relationships.

## CRediT authorship contribution statement

**Zahra Quettawala Mufaddal:** Writing – original draft, Visualization, Methodology, Formal analysis, Data curation, Conceptualization. **Rafay Khan:** Writing – original draft, Methodology, Formal analysis, Data curation, Conceptualization. **Omer Mustafa Siddiqui:** Writing – review & editing, Writing – original draft, Methodology, Data curation, Conceptualization. **Shanza Malik:** Writing – review & editing, Writing – original draft, Visualization, Data curation, Conceptualization. **Alina Baig:** Writing – review & editing, Writing – original draft, Methodology, Data curation, Conceptualization. **Mohammad Arham Siddiq:** Writing – review & editing, Writing – original draft, Visualization, Conceptualization. **Syed Husain Farhan:** Writing – review & editing, Writing – original draft, Visualization, Data curation. **Roha Saeed Memon:** Writing – review & editing, Writing – original draft, Visualization, Data curation. **Ishaque Hameed:** Writing – review & editing, Supervision, Methodology, Conceptualization. **Christopher J. Haas:** Writing – review & editing, Supervision, Methodology, Conceptualization. **Anita Tammara:** Writing – review & editing, Visualization, Supervision, Conceptualization.

## Declaration of competing interest

The authors declare that they have no known competing financial interests or personal relationships that could have appeared to influence the work reported in this paper.

## Appendix Supplementary materials

Image, application 1
