# Supplementary material for: Demographic and regional disparities in cancer cachexia-related mortality in the USA from 1999 to 2020 – A retrospective cross-sectional study
Source: Future Healthc J. 2025 Dec 10;13(1):100493. doi: 10.1016/j.fhj.2025.100493 (PMC12854984; doi:10.1016/j.fhj.2025.100493)
Supplement: Supplementary file 1 [file mmc1.docx]

**Demographic and Regional Disparities in Cancer Cachexia-Related Mortality in the United States from 1999 to 2020 – A Retrospective Cross-sectional Study**

**ABSTRACT**

**Background**

Nearly 2 million new cancer cases and over half a million cancer deaths in the United States were reported in 2024. Still there remains a paucity of data on cancer-cachexia–related mortality.

**Methods**

Centers for Disease Control and Prevention Wide-Ranging Online Data for Epidemiologic Research (CDC WONDER) database was utilized to identify cases where both cachexia and cancer were listed as either contributory or underlying causes of death between 1999 and 2020.

**Results**

Between 1999 and 2020, there were 64,106 deaths related to cancer and cachexia among adults aged ≥55 years. Overall, a declining trend has been identified from 1999 to 2019. Blacks had 1.4 times higher mortality than Whites. Rural dwellers had higher mortality than urban adults.. Lastly, states with AAMR in the 90th percentile (California, South Carolina, Utah, Georgia, Alaska and New Hampshire) displayed approximately 5-fold higher AAMR than states in the bottom 10th percentile.

**Conclusion**

Tailored public health strategies for healthcare access and risk factor management are needed to address these disparities.

**INTRODUCTION**

The National Center for Health Statistics projected almost 2 million new cancer cases and over half a million cancer deaths in the United States in 2024 [1]. Almost half of the cancer cases show a syndrome of cachexia characterized by anorexia and loss of adipose tissues and skeletal muscle mass [2]. In terms of diagnostic criteria, cachexia is defined as weight loss greater than 5%, weight loss greater than 2% in individuals already showing depletion according to current body weight and height (body-mass index [BMI] <20 kg/m^2^), or loss of skeletal muscle mass (sarcopenia) [3]. Cachexia, although recognized, is rarely assessed or actively managed, leading to increased morbidity and mortality. It is associated with reduced physical function, reduced tolerance to anticancer therapy, and reduced survival [4]. In one study, a reduced median survival of 13.6 months was observed in patients with overt weight loss as compared to 28.2 months in patients without weight loss [5]. Thus, identifying at-risk populations among patients with cancer and cachexia is crucial for timely intervention. Notably, studies have shown that the majority (75%) of individuals diagnosed with cancer cachexia were aged 56 and above [6]. In addition, according to SEER (surveillance, epidemiology, and end results program), over 80% of cancer diagnoses, and almost all (91.6%) of cancer-related deaths occur after age 55, with the median age at death ~73 years [7]. Therefore, we aim to provide a comprehensive analysis of mortality trends from 1999 to 2020 among elderly patients (≥55 years) with cancer cachexia, with a particular focus on identifying age, racial, and demographic subgroups disproportionately affected by cancer-cachexia–related mortality. This is intended to facilitate focused clinical approaches and evidence-based policy interventions to mitigate the effects such disparities.

**METHODS**

The CDC WONDER database was utilized to access data on cancer and cachexia-related mortality in the United States, from 1999 to 2020 [8]. The Multiple Cause-of-Death (MCOD) Public use death certificates were used to identify cases where both cachexia and cancer were listed as either contributory or underlying causes of death, on death-certificates. This database has previously been utilized to analyse mortality patterns related to cancer [9]. Cachexia was identified using the International Classification of Diseases 10th Revision (ICD-10) code R64, while cancer was identified using ICD-10 code C00-D48. We specifically focused on individuals ≥55 years because this age group accounts for >80% of cancer incidence and >90% of cancer-related mortality in the United States. This study was not subject to local Institutional Review Board approval as it utilized deidentified government-issued publicly available data and adhered to the STROBE (Strengthening the Reporting of Observational Studies in Epidemiology) guidelines for reporting (*Supplementary Table 1)*. We also studied cachexia-related mortality by cancer subtypes: gastrointestinal (GI) cancer (C15-C26), lung cancer (C30-C39), genitourinary (GU) cancer (C51-C58, C60-C68), prostate cancer (C61, D07.5, D29.1, D40.0), breast cancer (C50, D05), and brain cancer (C71, C72, D33, D43).

**Data Abstraction**

Data on primary outcomes such as overall cachexia and cancer-related deaths, population size, year, demographics, urban-rural classification, and states were extracted. In addition, data on secondary outcome (location of death and cancer subtypes) were also extracted. The location of death was categorized into three main groups: medical facilities, long-term residences, and other. Medical facilities comprised inpatient facilities, outpatient or ER facilities, cases of dead-on-arrival and those with unknown status in medical facilities. Long-term residence included descendant’s homes, hospice facilities and nursing homes/long term care facilities. Demographic information (sex, race/ethnicity, and age) and regional details (urban-rural classification and state) were extracted for the period spanning 1999 to 2020. To assess the population by urban-rural classification, the National Center for Health Statistics Urban-Rural Classification Scheme was employed, dividing counties into metropolitan (large central metropolitan, large fringe metropolitan, medium metropolitan, and small metropolitan) and nonmetropolitan (micropolitan and noncore) categories following the 2013 US census classification [10]. Race and ethnicity were classified into White, Black or African American (AA), Hispanic or Latino, American Indian (AI) or Alaskan Native (AN), and Asian or Pacific Islander (PI), based on data reported on death certificates, which has been used in previous analyses of the WONDER database. The regions were classified into Northeast, Midwest, South, and West based on Census Bureau definitions.

**Statistical Analysis**

To analyse national trends in cachexia and cancer-related mortality, we calculated the age-adjusted mortality rates (AAMRs) per 1,000,000 population from 1999 to 2020. AAMRs were stratified by gender, race, state, and metropolitan/ non-metropolitan status, along with 95% confidence intervals (CIs). AAMRs were calculated by standardizing cachexia and cancer-related deaths to the year 2000 U.S. standard population as previously described [11].

To determine the national annual trends in cachexia and cancer-related mortality, the Joinpoint Regression Program (Joinpoint V 5.2.0, National Cancer Institute) was used to identify the annual percent change (APC) with a 95% CI in AAMRs from 1999 to 2020 [12]. By fitting log-linear regression models to the data, this method identifies variations in AAMR over time, indicating increasing or decreasing trends in cachexia and cancer-related mortality. For heteroscedastic/ correlated error options, the standard error provided in the dataset was applied. We allowed a maximum of four joinpoints, and the optimal number of joinpoints was selected using the data-driven Bayesian Information Criterion (Weighted BIC). Joinpoints were restricted by grid search rules requiring at least two observations from a joinpoint to either end of the series, at least two observations between successive joinpoints, and testing only observed years as potential joinpoint locations (no interpolation between years). APCs and 95% CI for age-adjusted mortality rates were computed for the identified line segments connecting joinpoints. We used 2-tailed t-testing to determine if the slope of annual percent change describing the change in mortality was significantly different from zero. Statistical significance was set at P < 0.05.

**RESULTS**

**Overall cancer and cachexia-related deaths**

Between 1999 and 2020, there were 64,106 deaths related to cancer and cachexia among adults aged ≥55 years. Data on the location of death was available for 63,453 patients. Over two-thirds (71.3%) of these deaths occurred in long-term residences of patients; 37.5% in occurred in decedents’ homes (n=23,884), 28.9% in nursing homes or long-term care facilities (n=18,324), and 4.9% in hospice facilities (n=3,034). The remaining deaths were distributed between medical facilities (23.4%), comprising inpatient units (21.9%, n=13,921) and outpatient/ER settings (1.4%, n=908). 5.3% of total deaths occurred in other locations (n=3,382). *(Supplementary Table 2).* Of the total deaths attributed to cancer and cachexia, 84.8% occurred in White populations (n=53,749), while 12.6% occurred in Black/African American patients (n=8,102). The number of deaths was similar among males (52.9%) and females (47.1%) *(Supplementary Table 3)*.

The overall cancer and cachexia-related mortality approximately halved over the two decades (1999 AAMR: 65.8, 95% CI: 63.7 to 67.8; 2020 AAMR: 25.5, 95% CI: 24.5 to 26.6). This decline was characterized by three distinct periods of change. From 1999 to 2004, mortality rates decreased significantly with an APC of -6.7% (95% CI: -8.3 to -5.6); between 2004 and 2014, the decline slowed, with an APC of -2.8% (95% CI: -3.3 to -1.6); and from 2014 to 2020, the more steep decline in mortality was apparent, with an APC of -5.3% (95% CI: -7.1 to -4.3) *(Figure 1a).*

**Cancer and cachexia-related AAMR stratified by gender**

Throughout the study period, males had a consistently higher mortality rate than females, with an overall AAMR 1.6 times that of females (male AAMR: 51.3, 95% CI: 50.7 to 51.8; female AAMR: 32.3; 95% CI 31.9 to 32.6). Over the 21-year period, there was a consistent decline in AAMR in males, whereas AAMR in females decreased from 1999 to 2004 (APC: -5.8%, 95% CI: -7.6 to -4.7), followed by a period of stability until 2014. Thereafter, a rapid decline in mortality was noted from 2015 to 2020 (APC: -5.6%, 95% CI: -7.7 to -4.1) *(Supplementary Tables 4 and 5, Figure 1a)*.

# **Cancer and cachexia-related AAMR stratified by race**

Black/African American populations had AAMR almost 1.4 times higher than White populations. (Black/African American AAMR: 55, 95% CI: 53.7 to 56.2; White AAMR: 38.8, 95% CI: 38.4 to 39;). Black/African American population experienced a consistent decline in mortality (APC: -4.4%, 95% CI: -5.0 to -3.9), whereas White populations experienced a steady decline from 1999 to 2004 (APC: -7.2%, 95% CI: -11.2 to 5.6), followed by a plateauing of AAMRs during 2004-2008, and subsequently, a continued decrease from 2008 to 2020 (APC: -4.0%, 95% CI: -6.6 to-3.6) *(Supplementary Tables 4 and 5, Figure 1b)*.

**Cancer and cachexia-related AAMR stratified by region**

Overall, patients in non-metropolitan locations consistently had higher AAMRs than those in metropolitan locations throughout the study period. Mortality rate in non-metropolitan areas declined with an APC of -4.4% (95% CI-4.8 to -4.0), whereas patients in metropolitan areas had an initial decline in AAMR from 1999-2004 (APC: -7.0%, 95% CI: -10.3 to -5.3) after which no significant change was observed from 2004-2005, followed by a steady decline from 2015-2020 (APC: -5.7%, 95% CI-9.6 to -4) *(Supplementary Tables 5 and 6a, Figure 2a).*

States in the top 90th percentiles (California, South Carolina, Utah, Georgia, Alaska and New Hampshire), had approximately five times higher mortality rates than states in the lower 10th percentile (namely Louisiana, Mississippi, Arkansas, Kentucky, Massachusetts, Montana) *(Supplementary Table 6b, Figure 3b)*.

**Cancer and cachexia-related mortality stratified by cancer subtypes**

Throughout the study period, there was a steady decline in AAMRs. Mortality rates were highest due to cachexia and GI cancers (11.1, 95% CI:11 to 11.3), followed by cachexia and lung cancers (9.3; 95% CI: 9.2 to 9.5), cachexia and GU cancers (7.9; 95% CI: 7.8 to 8.1), cachexia and prostate cancers (3.7; 95% CI: 3.6 to 3.8), cachexia and breast cancers (2.9; 95% CI: 2.9 to 3) and finally, cachexia and brain cancers (1.5, 95% CI: 1.4 to 1.5). Notably, AAMR from GI cancers were 7.4 times that of brain cancers *(Supplementary Table 7)*. Mortality rates due to cachexia and GU cancer and cachexia and prostate cancer declined throughout the 2 decades, whereas those due to cachexia and lung, breast, and brain cancers have not decreased in the last decade after an initial steady decline *(Figure 3a).* Furthermore, mortality rates in males were higher for almost all cancer subtypes, except for breast cancer, in which females had a higher mortality, and brain cancer, in which mortality rates were equal *(Figure 3b).*

**DISCUSSION**

In this 20-year analysis of cancer-related cachexia mortality data from CDC WONDER, we report several key findings (see *central illustration*). First, the age-adjusted mortality rate amongst adults aged ≥55 years approximately halved over the last two decades. Second, the overall AAMR was higher among males, approximately 1.6 times compared to their female counterparts. Third, Black Americans had a consistently higher mortality than Whites. Lastly, significant regional variability existed, with states in the 90th percentile (California, South Carolina, Utah, Georgia, Alaska and New Hampshire) demonstrating a five-fold higher mortality rate compared with states in the 10th percentile (Louisiana, Mississippi, Arkansas, Kentucky, Massachusetts, Montana).

The American Cancer Society indicates that after an increasing trend in cancer-related mortality throughout the 20^th^ Century, since 1991, cancer-related mortality has been decreasing with an overall reduction of almost 31%. This decreasing trend is similar to the results seen in this study. These trends are attributed mainly to increased smoking cessation and improvements in early detection and treatment of cancers [13]. Our study demonstrates a higher cachexia-related mortality among cancer patients in the male population. Previous studies have demonstrated that male cancer patients have a higher incidence of cachexia with greater muscle loss and worse outcomes as compared to their female counterparts. Various mechanisms have been theorized, including sex differences in muscle fibre type and function, mitochondrial metabolism, global gene expression and signalling pathways, and regulatory mechanisms at the levels of sex chromosomes versus sex hormones. However, further research is required to fully understand the role of genders in susceptibility leading to muscle wasting and cachexia [14].

With respect to racial disparity, our study demonstrated 1.4 times the mortality among Blacks population as compared to Whites. Previous studies have demonstrated a similar outcome with respect to GI and Pancreatic cancers [15,16]. This difference could be partly explained due to the social determinants affecting Blacks. Prior studies have shown that Blacks are less likely to have routine follow-up with primary care providers and hence in part, plays a role in delayed detection of cancer [17].

Some cancer subtypes have been shown to be consistently associated with a higher risk of cancer-related malnutrition and cachexia. This is mainly due to cancer-induced activation of inflammatory pathways [18]. Our study demonstrated that GI and lung cancers had the highest cancer-cachexia related mortality among the cancer subtypes, a finding that aligns with the existing literature [19]. Gastroesophageal and pancreatic cancers can cause structural/obstructive changes, anorexia, and metabolic changes. Additionally, patients with pancreatic cancer may also have malabsorption due to pancreatic insufficiency thus causing severe weight loss in these two subtypes. Furthermore, the treatment itself causes major adverse effects leading to weight loss and cachexia due to dysphagia caused by radiation therapy, odynophagia due to chemotherapy, or decreased oral intake related to anorexia [20]. In regard to lung cancer, it has been observed that a weight loss ≥2% has been associated with poor overall and progression-free survival [21].

Mortality rates due to cachexia among lung, breast, and brain cancers remained stable in the past decade after an initial decline. A recent study published by Tan et al. demonstrated similar outcomes among malignant brain tumours. This could be explained by multiple factors. First, the observed trends in mortality could be explained by the development of improved diagnostic tests that may have missed earlier cases in the previous years. Second, glioblastoma is the most common brain tumour among adults aged 55 and older and exhibits the lowest survival rate due to its higher disease burden [22]. This, combined with the demographic shift towards an increasingly aging population, may contribute to the increased mortality trend seen in our study. In the population with breast cancer, no significant decline in mortality could be attributed to no major changes in the adjuvant standard of care (for adjuvant chemotherapy or hormone therapy) have taken place since 2010 [23,24].

A small retrospective study indicated that mean survival time was effectively longer (52 vs 23 days) in patient with cachexia with terminal cancer when appropriate nutritional intervention was initiated and therefore shows the significance of early recognition and treatment initiation for cancer related cachexia [25]. Transthyretin is a protein which has a rapid turnover of 2 days as compared to albumin (20 days) and is a well-known nutritional marker. Previously low TTR has been seen in patients with poor prognosis in cancer patients in palliative settings [26]. It has also been seen to rapidly increase in 4-8 days in malnourished children who were given adequate nutrition [27]. Therefore, further research can help in transthyretin or other biomarkers can help in nutritional assessment, prognosis and identification of responsive to nutrition in cancer cachexia  can be assessed.

This study further emphasizes the importance of cancer cachexia as also mentioned in the American society of clinical oncology guidelines that changes in body composition are associated with treatment toxicity, quality of life and survival. Such outcomes have initiated further research in developing biomarkers and research in therapy including effect of already easily available medications such as mirtazapine, olanzapine and anamorelin showing promise [28].

Our study has several limitations. First, as the CDC WONDER extracts information from death certificates, using ICD-codes to classify medical conditions carries a risk of inaccurate or missed diagnosis of cancer-related cachexia as a cause of death. This may lead to incorrect categorization, and one study demonstrated approximately 30% of incorrect classifications in the underlying cause of death [29]. Second, racial or ethnic categories can be incorrectly identified, and cause of death misclassified based on race, such as homicide victims being more commonly recorded as Black adults [30]. Third, our study targeted the older population, with middle-aged and young adults excluded from the analysis, which could alter the population-level results. In addition, we focused on Black and White adults as data regarding Hispanic or Latino, American Indian (AI) or Alaskan Native (AN), and Asian or Pacific Islander (PI) were either suppressed or unreliable. Fourth, information on cancer stage and treatment status was not available. Fifth, data available on the CDC WONDER database does not include details on social determinants of health that impact access to end-of-life care. These include patient diagnoses/co-morbidities, socioeconomic status, insurance coverage, prognosis, and preferences, along with factors such as the degree of support from caregivers and the rate of functional decline.

**CONCLUSION**

To summarize, cancer and cachexia-related mortality trends have steadily declined from 1999 to 2020. Significant disparities have been noted, with males faring worse than females and Black adults displaying worse outcomes than White adults. Furthermore, rural dwellers have poorer outcomes and require better access to healthcare to address such disparities. Targeted changes in policies should be made to address the underlying causes of such disparities, such as increasing the rate of healthcare utilization among males, diminishing financial and social barriers to equitable healthcare among all ethnicities/races, and increasing the number of specialized centers in rural areas. However, these patterns should be interpreted as correlations rather than causal relationships.

**DECLARATION OF COMPETING INTEREST**

The authors have nothing to declare.

Central Illustration


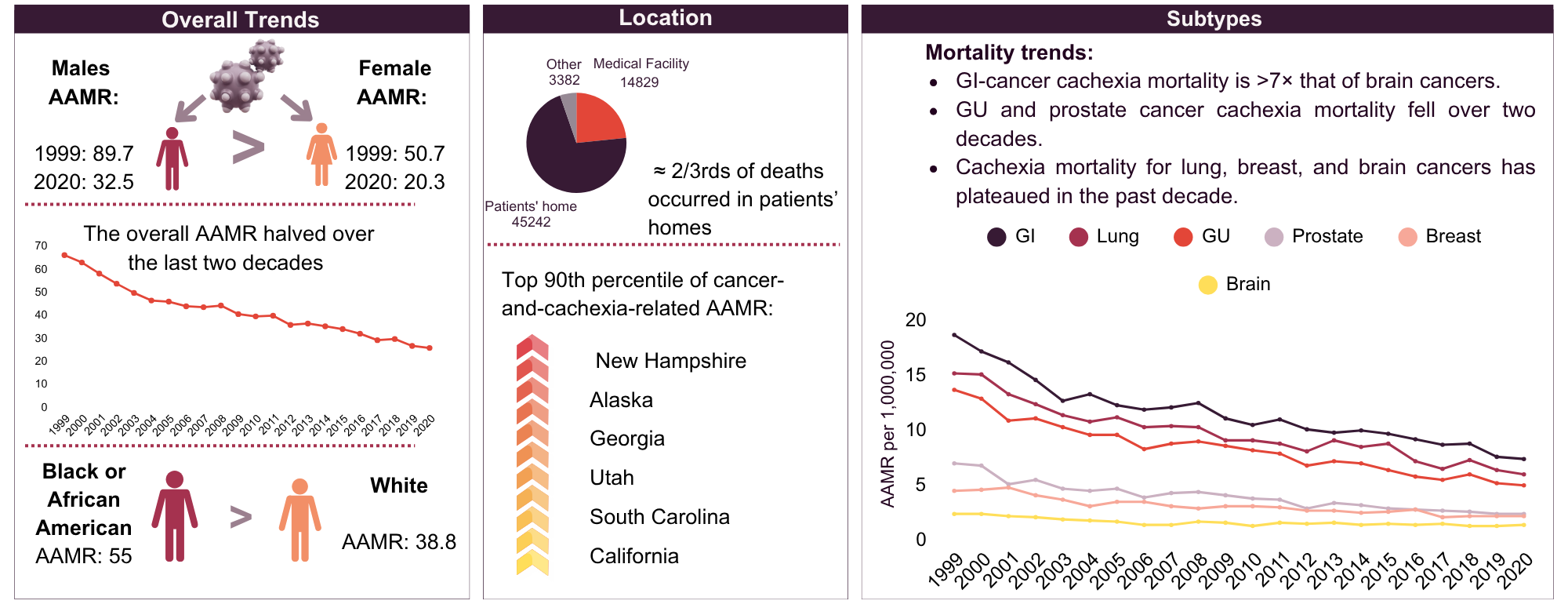


## **Figure 1a: Cancer and cachexia related Age-adjusted mortality rates in the overall population aged ≥55 years old and stratified by gender from 1999 - 2020**

## **Figure 1b: Cancer and cachexia related Age-adjusted mortality rates in the overall population aged ≥55 years old and stratified by race from 1999 – 2020**

## **Figure 2a: Cancer and cachexia related Age-adjusted mortality rates in population aged ≥55 years old stratified by urbanization from 1999 – 2020**

## **Figure 2b: Cancer and cachexia related Age-adjusted mortality rates in population aged ≥55 years old stratified by state from 1999 – 2020**


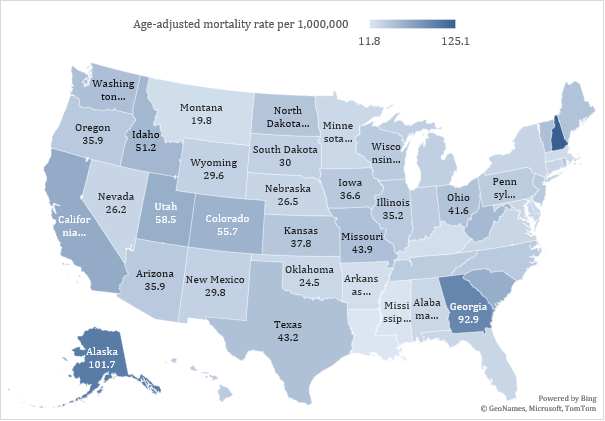


**Figure 3a: Cancer and cachexia related Age-adjusted mortality rates in the population aged ≥55 years old stratified by cancer subtypes from 1999 – 2020**

## **Figure 3b: Cancer and cachexia related total deaths in the population aged ≥55 years old stratified by cancer subtypes from 1999 – 2020**

## **Figure 3c: Age-adjusted mortality rate in the overall population aged ≥55 years, stratified by cancer and cachexia related death separately**

## **SUPPLEMENTAL FILE**

**Supplementary Table 1:** STROBE Checklist.

|  | **Item No** | **Recommendation** | **Page  No** |  |
| --- | --- | --- | --- | --- |
| Title and abstract | 1 | (a) Indicate the study’s design with a commonly used term in the title or the abstract | 2 |  |
|  |  | (b) Provide in the abstract an informative and balanced summary of what was done and what was found | 2 |  |
| **Introduction** | | | | |
| Background/rationale | 2 | Explain the scientific background and rationale for the investigation being reported | 3 |  |
| Objectives | 3 | State specific objectives, including any prespecified hypotheses | !!! |  |
| **Methods** | | | | |
| Study design | 4 | Present key elements of study design early in the paper | 3 - 4 |  |
| Setting | 5 | Describe the setting, locations, and relevant dates, including periods of recruitment, exposure, follow-up, and data collection | 3 |  |
| Participants | 6 | (a) Cohort study—Give the eligibility criteria, and the sources and methods of selection of participants. Describe methods of follow-up  Case-control study—Give the eligibility criteria, and the sources and methods of case ascertainment and control selection. Give the rationale for the choice of cases and controls  Cross-sectional study—Give the eligibility criteria, and the sources and methods of selection of participants | 2-3 |  |
|  |  | (b) Cohort study—For matched studies, give matching criteria and number of exposed and unexposed  Case-control study—For matched studies, give matching criteria and the number of controls per case | N/A |  |
| Variables | 7 | Clearly define all outcomes, exposures, predictors, potential confounders, and effect modifiers. Give diagnostic criteria, if applicable | Outcomes: 3-4  No exposures, predictors, confounders, or effect modifiers because our study is descriptive and ecological, not causal. |  |
| Data sources/ measurement | 8* | For each variable of interest, give sources of data and details of methods of assessment (measurement). Describe comparability of assessment methods if there is more than one group | 3-4  Because all variables were derived from a single national mortality reporting system using uniform coding standards, assessment methods were fully comparable across demographic and geographic subgroups |  |
| Bias | 9 | Describe any efforts to address potential sources of bias | 5 |  |
| Study size | 10 | Explain how the study size was arrived at | N/A.  The study included all eligible deaths recorded in the national CDC WONDER MCOD database between 1999 and 2020. No sample size was calculated. |  |
| Quantitative variables | 11 | Explain how quantitative variables were handled in the analyses. If applicable, describe which groupings were chosen and why | 3-4 |  |
| Statistical methods | 12 | (a) Describe all statistical methods, including those used to control for confounding | 3-4 |  |
|  |  | (b) Describe any methods used to examine subgroups and interactions | Page 4  No interaction tests were conducted, because we used population-level data, and not individual-level data. Therefore, covariates were not used, and no regression models were built. |  |
|  |  | (c) Explain how missing data were addressed | 3 |  |
|  |  | (d) Cohort study—If applicable, explain how loss to follow-up was addressed  Case-control study—If applicable, explain how matching of cases and controls was addressed  Cross-sectional study—If applicable, describe analytical methods taking account of sampling strategy | N/A Because the dataset includes the entire population rather than a sample, no sampling weights, cluster adjustments, or survey-specific analytical methods were required. |  |
|  |  | (e) Describe any sensitivity analyses | N/A  Because our analysis relied on complete population-level mortality data and did not involve assumptions, sensitivity analyses were not indicated. |  |
| **Results** | | | |  |
| Participants | 13* | (a) Report numbers of individuals at each stage of study—e.g. numbers potentially eligible, examined for eligibility, confirmed eligible, included in the study, completing follow-up, and analysed | N/A  Our study used publicly available data. No participants were enrolled, and consequently, no staged eligibility assessment or follow-up was applicable. |  |
|  |  | (b) Give reasons for non-participation at each stage |  |  |
|  |  | (c) Consider use of a flow diagram |  |  |
| Descriptive data | 14* | (a) Give characteristics of study participants (ego demographic, clinical, social) and information on exposures and potential confounders | 2- 4 |  |
|  |  | (b) Indicate number of participants with missing data for each variable of interest | 9 |  |
|  |  | (c) Cohort study—Summarise follow-up time (e.g., average and total amount) | N/A |  |
| Outcome data | 15* | Cohort study—Report numbers of outcome events or summary measures over time | N/A |  |
|  |  | Case-control study—Report numbers in each exposure category, or summary measures of exposure | N/A |  |
|  |  | Cross-sectional study—Report numbers of outcome events or summary measures | 5 |  |
| Main results | 16 | (a) Give unadjusted estimates and, if applicable, confounder-adjusted estimates and their precision (ego, 95% confidence interval). Make clear which confounders were adjusted for and why they were included | 4-6  No confounder-adjusted estimates were applicable due to the absence of individual-level covariates in CDC WONDER. |  |
|  |  | (b) Report category boundaries when continuous variables were categorized | 4 |  |
|  |  | (c) If relevant, consider translating estimates of relative risk into absolute risk for a meaningful time period | N/A.  Because the study reports age-adjusted mortality rates rather than relative risks, translation of relative risks into absolute risks was not applicable. |  |
| Other analyses | 17 | Report other analyses done—e.g. analyses of subgroups and interactions, and sensitivity analyses | 4 - 6  We did not test interactions because CDC WONDER lacks the individual-level variables needed for such analyses. |  |
| **Discussion** | | | |  |
| Key results | 18 | Summarise key results with reference to study objectives | 7 |  |
| Limitations | 19 | Discuss limitations of the study, taking into account sources of potential bias or imprecision. Discuss both direction and magnitude of any potential bias | 9 |  |
| Interpretation | 20 | Give a cautious overall interpretation of results considering objectives, limitations, multiplicity of analyses, results from similar studies, and other relevant evidence | 9 |  |
| Generalisability | 21 | Discuss the generalisability (external validity) of the study results | 9 |  |
| **Other information** | | | |  |
| Funding | 22 | Give the source of funding and the role of the funders for the present study and, if applicable, for the original study on which the present article is based | 9 |  |

## **Supplementary Table 2: Cancer and Cachexia Related Deaths for among the population aged ≥55 years, stratified by Location of Death in the United States, 1999-2020**

| **Year** | **Medical Facility - Inpatient** | **Medical Facility - Outpatient or ER** | **Decedent's home** | **Hospice facility** | **Nursing home/long term care** | **Other** |
| --- | --- | --- | --- | --- | --- | --- |
| 1999 | 980 | 49 | 1391 | Missing | 1173 | 200 |
| 2000 | 911 | 45 | 1361 | Missing | 1168 | 180 |
| 2001 | 863 | 48 | 1200 | Missing | 1138 | 189 |
| 2002 | 849 | 59 | 1094 | Missing | 1028 | 196 |
| 2003 | 753 | 43 | 1097 | Suppressed | 960 | 182 |
| 2004 | 636 | 31 | 1103 | 19 | 951 | 164 |
| 2005 | 692 | 43 | 1075 | 54 | 879 | 186 |
| 2006 | 694 | 49 | 991 | 59 | 906 | 142 |
| 2007 | 686 | 30 | 1037 | 123 | 866 | 153 |
| 2008 | 654 | 39 | 1007 | 190 | 845 | 120 |
| 2009 | 551 | 42 | 917 | 164 | 779 | 135 |
| 2010 | 520 | 41 | 1023 | 216 | 854 | 146 |
| 2011 | 555 | 37 | 1037 | 239 | 897 | 144 |
| 2012 | 516 | 45 | 1008 | 247 | 759 | 122 |
| 2013 | 544 | 47 | 1077 | 156 | 756 | 224 |
| 2014 | 528 | 37 | 1081 | 281 | 707 | 138 |
| 2015 | 516 | 30 | 1119 | 280 | 722 | 93 |
| 2016 | 563 | 33 | 1020 | 228 | 698 | 130 |
| 2017 | 528 | 44 | 1012 | 180 | 618 | 127 |
| 2018 | 509 | 40 | 1109 | 197 | 599 | 147 |
| 2019 | 476 | 39 | 1002 | 209 | 540 | 130 |
| 2020 | 397 | 37 | 1123 | 192 | 481 | 134 |
| **Total** | **13921** | **908** | **23884** | **3034** | **18324** | **3382** |

##

## **Supplementary Table 3: Cancer and Cachexia Related Deaths among the population aged ≥ 55 years, stratified by gender and race, in the United States, 1999-2020**

| **Year** | **Overall** | **Women** | **Men** | **Black or African American** | **White** |
| --- | --- | --- | --- | --- | --- |
| 1999 | 3836 | 1791 | 2045 | 446 | 3304 |
| 2000 | 3693 | 1701 | 1992 | 437 | 3174 |
| 2001 | 3467 | 1712 | 1755 | 428 | 2957 |
| 2002 | 3253 | 1584 | 1669 | 422 | 2731 |
| 2003 | 3061 | 1489 | 1572 | 370 | 2600 |
| 2004 | 2912 | 1410 | 1502 | 335 | 2493 |
| 2005 | 2940 | 1406 | 1534 | 358 | 2494 |
| 2006 | 2865 | 1390 | 1475 | 365 | 2414 |
| 2007 | 2903 | 1372 | 1531 | 358 | 2453 |
| 2008 | 3009 | 1434 | 1575 | 370 | 2545 |
| 2009 | 2830 | 1349 | 1481 | 372 | 2355 |
| 2010 | 2810 | 1327 | 1483 | 318 | 2381 |
| 2011 | 2916 | 1379 | 1537 | 379 | 2423 |
| 2012 | 2703 | 1270 | 1433 | 329 | 2262 |
| 2013 | 2812 | 1317 | 1495 | 369 | 2328 |
| 2014 | 2779 | 1273 | 1506 | 412 | 2254 |
| 2015 | 2764 | 1245 | 1519 | 382 | 2267 |
| 2016 | 2674 | 1244 | 1430 | 368 | 2180 |
| 2017 | 2513 | 1156 | 1357 | 326 | 2078 |
| 2018 | 2604 | 1187 | 1417 | 324 | 2151 |
| 2019 | 2397 | 1089 | 1308 | 312 | 1977 |
| 2020 | 2365 | 1061 | 1304 | 322 | 1928 |
| **Total** | **64106** | **30186** | **33920** | **8102** | **53749** |

## **Supplementary Table 4: Cancer and Cachexia Related Age-adjusted mortality rates per 1,000,000 among the overall population aged ≥55 and stratified by gender and race in the United States, 1999-2020**

| **Year** | **Overall** | **Women** | **Men** | **Black or African American** | **White** |
| --- | --- | --- | --- | --- | --- |
| 1999 | 65.8  (63.7 – 67.8) | 50.7 (48.3 - 53.1) | 89.7 (85.7 - 93.6) | 91.4 (82.8 - 99.9) | 63.6  (61.4 - 65.7) |
| 2000 | 62.6  (60.5 - 64.6) | 47.6  (45.3 - 49.9) | 86  (82.2 - 89.9) | 89.2 (80.8 - 97.6) | 60.5  (58.4 - 62.6) |
| 2001 | 57.8  (55.9 - 59.8) | 47.2  (45 - 49.4) | 74.3  (70.8 - 77.8) | 85.1 (76.9 - 93.2) | 55.6  (53.6 - 57.6) |
| 2002 | 53.4  (51.6 - 55.2) | 43  (40.9 - 45.2) | 69.8  (66.4 - 73.2) | 82.2  (74.3 - 90.2) | 50.5  (48.6 - 52.4) |
| 2003 | 49.4  (47.6 - 51.1) | 40  (38 - 42.1) | 64.2  (60.9 - 67.4) | 71.4 (64.1 - 78.8) | 47.3  (45.5 - 49.2) |
| 2004 | 46.1 (44.5 - 47.8) | 37.5  (35.5 - 39.5) | 59.6  (56.5 - 62.7) | 63.2  (56.3 - 70) | 44.8  (43 - 46.5) |
| 2005 | 45.6  (44 - 47.3) | 36.5  (34.6 - 38.4) | 59.4  (56.4 - 62.4) | 64.2  (57.4 - 70.9) | 44  (42.2 - 45.7) |
| 2006 | 43.6  (42 - 45.2) | 35.5  (33.6 - 37.3) | 55.6  (52.7 - 58.5) | 64.4  (57.7 - 71.1) | 41.8  (40.1 - 43.5) |
| 2007 | 43.2  (41.6 - 44.8) | 34.4  (32.6 - 36.3) | 56.6  (53.7 - 59.4) | 61.1  (54.7 - 67.6) | 41.7  (40 - 43.3) |
| 2008 | 43.9 (42.4 - 45.5) | 35.6  (33.7 - 37.4) | 56  (53.2 - 58.9) | 61  (54.6 - 67.3) | 42.5  (40.9 - 44.2) |
| 2009 | 40.2 (38.7 - 41.7) | 32.4  (30.7 - 34.2) | 51.4  (48.7 - 54.1) | 58.6  (52.5 - 64.7) | 38.4  (36.8 - 39.9) |
| 2010 | 39.2  (37.7 - 40.6) | 31.5  (29.8 - 33.2) | 50.7  (48 - 53.3) | 49.1  (43.5 - 54.6) | 38.1  (36.6 - 39.7) |
| 2011 | 39.5  (38.1 - 41) | 31.7  (30 - 33.4) | 50.4  (47.9 - 53) | 55.8  (50 - 61.6) | 37.8  (36.3 - 39.4) |
| 2012 | 35.5  (34.2 - 36.9) | 28.7  (27.1 - 30.3) | 45.2  (42.8 - 47.6) | 45.9  (40.8 - 51) | 34.5  (33 - 35.9) |
| 2013 | 36.1  (34.7 - 37.4) | 29.2  (27.6 - 30.8) | 45.5  (43.1 - 47.8) | 49.5  (44.3 - 54.7) | 34.7  (33.3 - 36.1) |
| 2014 | 34.9  (33.5 - 36.2) | 27.6  (26.1 - 29.2) | 44.7  (42.4 - 47) | 53.6  (48.2 - 59) | 33  (31.6 - 34.4) |
| 2015 | 33.7  (32.5 - 35) | 26.5  (25 - 28) | 43.7  (41.5 - 45.9) | 48.6  (43.5 - 53.6) | 32.4  (31.1 - 33.8) |
| 2016 | 31.7  (30.5 - 33) | 25.7  (24.2 - 27.1) | 39.8  (37.7 - 42) | 44.5  (39.8 - 49.2) | 30.4  (29.1 - 31.7) |
| 2017 | 28.9  (27.7 - 30) | 23.4  (22.1 - 24.8) | 36.3  (34.3 - 38.2) | 37.2  (33 - 41.4) | 28.2  (26.9 - 29.4) |
| 2018 | 29.4  (28.2 - 30.5) | 23.6  (22.3 - 25) | 37.2  (35.2 - 39.2) | 36.6  (32.5 - 40.7) | 28.7  (27.5 - 29.9) |
| 2019 | 26.4  (25.4 - 27.5) | 21.3  (20 - 22.6) | 33.4  (31.6 - 35.3) | 32.9  (29.1 - 36.7) | 25.9  (24.8 - 27.1) |
| 2020 | 25.5  (24.5 - 26.6) | 20.3  (19.1 - 21.5) | 32.5  (30.7 - 34.3) | 33.5  (29.7 - 37.3) | 24.8  (23.7 - 25.9) |
| **total** | **40.1**  **(39.8 40.4)** | **32.3**  **(31.9 - 32.6)** | **51.3**  **(50.7 - 51.8)** | **55**  **(53.7 - 56.2)** | **38.8**  **(38.4 - 39.1)** |

## **Supplementary Table 5: Annual percentage change (APC) of Cancer and cachexia-related AAMR in the overall population aged ≥55 years and stratified by gender, race and metro/non-metro status in the United States, 1999-2020**

| **Cohort** | **Lower Endpoint** | **Upper Endpoint** | **APC** | **APC 95% Lower CI** | **APC 95% Upper CI** | **P-Value** |
| --- | --- | --- | --- | --- | --- | --- |
| Overall | 1999 | 2004 | -6.8 | -8.3 | -5.6 | 0* |
| Overall | 2004 | 2014 | -2.8 | -3.3 | -1.6 | 0.008398* |
| Overall | 2014 | 2020 | -5.3 | -7.1 | -4.3 | 0* |
| Black or African American | 1999 | 2020 | -4.4 | -5.0 | -3.9 | 0* |
| White | 1999 | 2004 | -7.2 | -11.2 | -5.6 | 0* |
| White | 2004 | 2008 | -1.5 | -3.4 | 1.4 | 0.240352 |
| White | 2008 | 2020 | -4.0 | -6.6 | -3.6 | 0.0008* |
| Female | 1999 | 2004 | -5.8 | -7.6 | -4.7 | 0* |
| Female | 2004 | 2014 | -3.0 | -3.4 | -1.1 | 0.005199* |
| Female | 2014 | 2020 | -5.1 | -7.7 | -4.1 | 0* |
| Male | 1999 | 2003 | -8.6 | -12.7 | -6.4 | 0* |
| Male | 2003 | 2015 | -3.2 | -3.6 | -0.1 | 0.045991* |
| Male | 2015 | 2020 | -5.6 | -9.3 | -4.2 | 0* |
| Metro | 1999 | 2004 | -7.0 | -10.3 | -5.3 | 0* |
| Metro | 2004 | 2015 | -2.6 | -3.2 | 0.1 | 0.057588 |
| Metro | 2015 | 2020 | -5.7 | -9.6 | -4.1 | 0* |
| Non metro | 1999 | 2020 | -4.4 | -4.8 | -4.0 | 0* |

## **Supplementary Table 6a: Cancer and Cachexia Related Age-adjusted mortality rates per 1,000,000 stratified by urbanisation among the older population (≥55 years) in the United States, 1999-2020**

| **Year** | **Metro/Urban** | **Non-metro/rural** |
| --- | --- | --- |
| 1999 | 63.8  (61.5-66) | 74.3  (69.3-79.4) |
| 2000 | 60.6  (58.4-62.8) | 71  (66-75.9) |
| 2001 | 55.6  (53.5-57.7) | 67.5  (62.7-72.3) |
| 2002 | 50.8  (48.8-52.8) | 64.8  (60.1-69.4) |
| 2003 | 47.7  (45.8-49.6) | 56.9  (52.6-61.2) |
| 2004 | 43.4  (41.6-45.2) | 58.2  (53.8-62.5) |
| 2005 | 44.3  (42.5-46.1) | 51.4  (47.4-55.5) |
| 2006 | 41.6  (39.9-43.4) | 52.3  (48.3-56.4) |
| 2007 | 42.2  (40.5-43.9) | 47.8  (43.9-51.7) |
| 2008 | 43  (41.3-44.7) | 48.1  (44.2-52) |
| 2009 | 38.7  (37.1-40.3) | 47  (43.2-50.8) |
| 2010 | 37.6  (36-39.2) | 46.4  (42.6-50.1) |
| 2011 | 38.2  (36.6-39.7) | 45.6  (41.9-49.30 |
| 2012 | 33.6  (32.1-35) | 44.9  (41.3-48.5) |
| 2013 | 35.1  (33.7-36.6) | 40.7  (37.2-44.1) |
| 2014 | 34.9  (33.4-36.3) | 34.9  (31.7-38) |
| 2015 | 33.8  (32.4-35.2) | 33.5  (30.4-36.5) |
| 2016 | 31.4  (30-32.7) | 33.6  (30.6-36.6) |
| 2017 | 28.4  (27.1-29.6) | 31.6  (28.7-34.5) |
| 2018 | 28.5  (27.3-29.8) | 33.4  (30.5-36.4) |
| 2019 | 26.1  (25-27.3) | 28.1  (25.4-30.8) |
| 2020 | 25.2  (24-26.3) | 27.7  (25.1-30.3) |
| Total | 38.8  (38.4-39.1) | 46  (45.2-46.8) |

## **Supplementary Table 6b: Cancer and Cachexia Related Age-adjusted mortality rates per 1,000,000 stratified by state among the older population (≥55 years) in the United States, 1999-2020**

| **State** | **RANK** | **Percentile** | **AAMR** **(95% CI)** |
| --- | --- | --- | --- |
| Louisiana | 1 | 2 | 11.8  (14.9-13.3) |
| Mississippi | 2 | 4 | 13.9  (17.9-15.9) |
| Arkansas | 3 | 6 | 18.3  (22.8-20.5) |
| Kentucky | 4 | 8 | 18.9  (22.7-20.8) |
| Massachusetts | 5 | 10 | 19.8  (27.8-23.8) |
| Montana | 6 | 12 | 21.3  (24.4-22.9) |
| Minnesota | 7 | 14 | 23.3  (27.1-25.2) |
| Florida | 8 | 16 | 23.3  (32.6-27.9) |
| Alabama | 9 | 18 | 24.5  (29.1-26.8) |
| Oklahoma | 10 | 20 | 24.5  (28.5-26.5) |
| Virginia | 11 | 22 | 25.5  (27.3-26.4) |
| Maryland | 12 | 24 | 25.6  (28.9-27.2) |
| Delaware | 13 | 25 | 25.7  (29.6-27.6) |
| New York | 14 | 27 | 25.9  (30.5-28.2) |
| Connecticut | 15 | 29 | 26.2  (32.4-29.3) |
| Nevada | 16 | 31 | 26.5  (33.3-29.9) |
| Nebraska | 17 | 33 | 27.1  (29.2-28.1) |
| Michigan | 18 | 35 | 29.6  (44-36.8) |
| New Mexico | 19 | 37 | 29.8  (36.9-33.3) |
| Indiana | 20 | 39 | 30  (40.7-35.4) |
| South Dakota | 20 | 39 | 31.2  (34.2-32.7) |
| Pennsylvania | 22 | 43 | 33.4  (37.4-35.4) |
| Tennessee | 23 | 45 | 33.6  (48.6-41.1) |
| Illinois | 24 | 47 | 34  (38.1-36.1) |
| Wyoming | 25 | 49 | 34  (42.7-38.3) |
| Wisconsin | 26 | 51 | 34.2  (36.8-35.5) |
| New Jersey | 27 | 53 | 34.7  (39-36.9) |
| North Carolina | 27 | 53 | 35.2  (38.1-36.7) |
| Arizona | 29 | 57 | 35.3  (38.8-37.1) |
| Hawaii | 30 | 59 | 35.4  (38.9-37.1) |
| Oregon | 31 | 61 | 35.9  (41.2-38.6) |
| Iowa | 32 | 63 | 35.9  (40-37.9) |
| Kansas | 33 | 65 | 36.6  (42.4-39.5) |
| District of Columbia | 34 | 67 | 37.8  (44.3-41) |
| Rhode Island | 35 | 69 | 37.9  (48.1-43) |
| Ohio | 36 | 71 | 39.6  (53.2-46.4) |
| Texas | 37 | 73 | 41.2  (50.5-45.8) |
| Maine | 38 | 75 | 41.6  (44.8-43.2) |
| Missouri | 39 | 76 | 43.2  (45.8-44.5) |
| North Dakota | 40 | 78 | 43.9  (48.6-46.3) |
| Washington | 41 | 80 | 44  (58.8-51.4) |
| Vermont | 42 | 82 | 44.5  (49.2-46.8) |
| West Virginia | 43 | 84 | 49.9  (58.5-54.2) |
| Idaho | 44 | 86 | 51.2  (62-56.6) |
| Colorado | 45 | 88 | 55.7  (62.1-58.9) |
| Lungs | 46 | 90 | 58.5  (68.6-63.6) |
| South Carolina | 46 | 90 | 59.3  (65.7-62.5) |
| Utah | 48 | 94 | 61.3  (63.6-62.5) |
| Georgia | 49 | 96 | 92.9  (98.9-95.9) |
| Alaska | 50 | 98 | 101.7  (132.8-117.3) |
| New Hampshire | 51 | 100 | 125.1  (142.2-133.7) |

# **Supplementary Table 7: Cancer and Cachexia Related Age-adjusted mortality rates per 1,000,000 stratified by cancer subtypes among the older population (≥55 years) in the United States, 1999-2020**

| **Year** | **Lungs** | **GI** | **Breast** | **Brain** | **Prostate** | **GU** |
| --- | --- | --- | --- | --- | --- | --- |
| 1999 | 10.7  (9.6-11.8) | 18.6  (17.5-19.7) | 4.4  (3.9-4.9) | 2.3  (1.9-2.7) | 6.9  (6.2-7.5) | 13.6  (12.7-14.6) |
| 2000 | 15  (14.0-16.0) | 17.1  (16-18.1) | 4.5  4-5.1) | 2.3  (2-2.7) | 6.7  (6.1-7.4) | 12.8  (11.9-13.7) |
| 2001 | 13.2  (12.3-14.2) | 16.1  (15.1-17.1) | 4.7  (4.2-5.3) | 2.1  (1.7-2.5) | 5  (4.4-5.6) | 10.8  (10-11.6) |
| 2002 | 12.3  (11.5-13.2) | 14.5  (13.6-15.5) | 4  (3.5-4.5) | 2  (1.6-2.3) | 5.4  (4.8-6) | 11  (10.2-11.8) |
| 2003 | 11.3  (10.4-12.1) | 12.6  (11.7-13.5) | 3.6  (3.1-4) | 1.8  (1.4-2.1) | 4.6  (4.1-5.2) | 10.2  (9.4-11) |
| 2004 | 10.7  (9.9-11.5) | 13.2  (12.3-14) | 3  (2.6-3.4) | 1.7  (1.3-2) | 4.4  (3.9-4.9) | 9.5  (8.8-10.3) |
| 2005 | 11.1  (10.3-11.9) | 12.2  (11.3-13) | 3.4  (3-3.9) | 1.6  (1.3-1.9) | 4.6  (4.1-5.2) | 9.5  (8.7-10.3) |
| 2006 | 10.2  (9.4-11) | 11.8  (10.9-12.6) | 3.4  (2.9-3.8) | 1.3  (1.1-1.6) | 3.8  (3.3-4.2) | 8.2  (7.5-8.9) |
| 2007 | 10.3  (9.5-11) | 12  (11.2-12.8) | 3  (2.6-3.4) | 1.3  (1-1.6) | 4.2  (3.7-4.7 | 8.7  (8-9.4) |
| 2008 | 10.2  (9.5-11) | 12.4  (11.6-13.3) | 2.8  (2.4-3.2) | 1.6  (1.3-1.9) | 4.3  (3.8-4.7) | 8.9  (8.2-9.6) |
| 2009 | 9  (8.3-9.7) | 11  (10.2-11.8) | 3  (2.5-3.4) | 1.5  (1.2-1.8) | 4  (3.5-4.5) | 8.5  (7.8-9.2) |
| 2010 | 9  (8.3-9.7) | 10.4  (9.6 -11.1) | 3  (2.6-3.4) | 1.2  (0.9-1.5) | 3.7  (3.3-4.2) | 8.1  (7.4-8.7) |
| 2011 | 8.7  (8-9.4) | 10.9  (10.2-11.7) | 2.9  (2.5-3.3) | 1.5  (1.2-1.7) | 3.6  (3.2-4) | 7.8  (7.2-8.5) |
| 2012 | 8  (7.4-8.6) | 10  (9.3-10.7) | 2.6  (2.2-2.9) | 1.4  (1.1-1.6) | 2.8  (2.4-3.2) | 6.7  (6.1-7.3) |
| 2013 | 9  (8.3-9.7) | 9.7  (9-10.4) | 2.6  (2.2-2.9) | 1.5  (1.2-1.7) | 3.3  (2.8-3.7) | 7.1  (6.5-7.7) |
| 2014 | 8.4  (7.7-9) | 9.9  (9.2-10.5) | 2.4  (2.1-2.7) | 1.3  (1.1-1.6) | 3.1  (2.7-3.5) | 6.9  (6.3-7.5) |
| 2015 | 8.7  (8.1-9.4) | 9.6  (8.9-10.3) | 2.5  (2.1-2.8) | 1.4  (1.2-1.7) | 2.8  (2.4-3.2) | 6.3  (5.7-6.8) |
| 2016 | 7.1  (6.6-7.7) | 9.1  (8.5-9.8) | 2.7  (2.3-3) | 1.3  (1.1-1.6) | 2.7  (2.3-3) | 5.7  (5.2-6.3) |
| 2017 | 6.4  (5.9-7) | 8.6  (8-9.2) | 2  (1.7-2.3) | 1.4  (1.2-1.7) | 2.6  (2.2-2.9) | 5.4  (4.9-5.8) |
| 2018 | 7.2  (6.6-7.8) | 8.7  (8.1-9.3) | 2.1  (1.8-2.4) | 1.2  (1-1.5) | 2.5  (2.2-2.8) | 5.9  (5.3-6.4) |
| 2019 | 6.3  (5.8-6.9) | 7.5  (6.9-8) | 2.1  (1.8-2.4) | 1.2  (1-1.4) | 2.3  (2-2.6) | 5.1  (4.6-5.5) |
| 2020 | 5.9  (5.4-6.4) | 7.3  (6.7-7.8) | 2.1  (1.8-2.4) | 1.3  (1-1.5) | 2.3  (2-2.6) | 4.9  (4.5-5.4) |
| **Total** | **9.3**  **(9.2-9.5)** | **11.1**  **(11-11.3)** | **2.9**  **(2.9-3)** | **1.5**  **(1.4-1.5)** | **3.7**  **(3.6-3.8)** | **7.9**  **(7.8-8.1)** |

## **Supplementary Table 8: Cancer and Cachexia Related Annual percentage change (APC) the overall population aged ≥55 years and stratified by gender, race and metro/non-metro status in the United States, 1999-2020**

| **Cohort** | **Lower Endpoint** | **Upper Endpoint** | **APC** | **APC 95% Lower CI** | **APC 95% Upper CI** | **P-Value** |
| --- | --- | --- | --- | --- | --- | --- |
| GI | 1999 | 2003 | -8.3 | -14.0 | -4.8 | 0.0008* |
| GI | 2003 | 2018 | -2.8 | -3.3 | 2.0 | 0.186363 |
| GI | 2018 | 2020 | -9.0 | -15.6 | -3.2 | 0.0004* |
| Lung | 1999 | 2003 | -7.0 | -13.4 | -3.5 | 0.013597* |
| Lung | 2003 | 2020 | -3.3 | -7.9 | 0.9 | 0.065187 |
| GU | 1999 | 2020 | -4.3 | -4.9 | -3.7 | 0* |
| Prostate | 1999 | 2001 | -13.6 | -18.4 | -4.7 | 0.0004* |
| Prostate | 2001 | 2020 | -4.3 | -10.6 | -0.3 | 0.044791* |
| Breast | 1999 | 2004 | -6.6 | -14.1 | -3.4 | 0.013597* |
| Breast | 2004 | 2020 | -3.0 | -5.6 | 2.4 | 0.09998 |
| Brain | 1999 | 2006 | -7.1 | -17.7 | -3.8 | 0* |
| Brain | 2006 | 2020 | -0.7 | -1.8 | 1.6 | 0.393121 |

##

## **Supplementary Table 9a: Age-adjusted mortality rate in the overall population aged ≥55 years, stratified by cancer and cachexia related death separately**

| **Year** | **Cancer** | **Cachexia** |
| --- | --- | --- |
| 1999 | 9,472.4  (9,447.5 - 9,497.4) | 194.1  (190.5 - 197.7) |
| 2000 | 9,410.1  (9,385.4 - 9,434.8) | 192.6  (189.0 - 196.1) |
| 2001 | 9,237.7  (9,213.3 - 9,262.0) | 185.1  (181.6 - 188.5) |
| 2002 | 9,130.8  (9,106.8 - 9,154.8) | 179.5  (176.1 - 182.8) |
| 2003 | 8,962.4  (8,938.9 - 8,986.0) | 173.6  (170.3 - 176.9) |
| 2004 | 8,763.9  (8,740.7 - 8,787.0) | 163.1  (159.9 - 166.2) |
| 2005 | 8,675.5  (8,652.7 - 8,698.3) | 158.7  (155.6 - 161.8) |
| 2006 | 8,515.6  (8,493.2 - 8,538.0) | 160.8  (157.7 - 163.9) |
| 2007 | 8,389.6  (8,367.6 - 8,411.6) | 164.2  (161.1 - 167.3) |
| 2008 | 8,260.5  (8,238.9 - 8,282.1) | 160  (157.0 - 163.0) |
| 2009 | 8,084.8  (8,063.7 - 8,106.0) | 150.8  (147.9 - 153.7) |
| 2010 | 8,070.1  (8,049.1 - 8,091.1) | 156.8  (153.9 - 159.7) |
| 2011 | 7,865.2  (7,844.7 - 7,885.6) | 153.9  (151.1 - 156.8) |
| 2012 | 7,754.1  (7,734.1 - 7,774.2) | 149.4  (146.6 - 152.2) |
| 2013 | 7,598.8  (7,579.3 - 7,618.4) | 149.8  (147.1 - 152.5) |
| 2014 | 7,505.9  (7,486.7 - 7,525.1) | 140.3  (137.7 - 143.0) |
| 2015 | 7,400.6  (7,381.8 - 7,419.4) | 141.8  (139.2 - 144.4) |
| 2016 | 7,285.8  (7,267.3 - 7,304.3) | 132.2  (129.7 - 134.7) |
| 2017 | 7,173.3  (7,155.3 - 7,191.4) | 130.6  (128.1 - 133.0) |
| 2018 | 7,045.1  (7,027.5 - 7,062.7) | 125  (122.7 - 127.4) |
| 2019 | 6,928.2  (6,911.0 - 6,945.5) | 111.8  (109.6 - 114.0) |
| 2020 | 7,039.2  (7,022.0 - 7,056.5) | 115.4  (113.2 - 117.7) |
| **Total** | **7,999.1**  **(7,994.7 - 8,003.5)** | **151.2**  **(150.6 - 151.8)** |

## **Supplementary Table 9b: Annual percentage change in mortality among the overall population aged ≥55 years, stratified by cancer and cachexia related death separately**

| **Cohort** | **Lower Endpoint** | **Upper Endpoint** | **APC** | **APC 95% Lower CI** | **APC 95% Upper CI** | **P-Value** |
| --- | --- | --- | --- | --- | --- | --- |
| Cancer | 1999 | 2018 | -1.6 | -1.6 | -1.5 | 0 |
| Cancer | 2018 | 2020 | -0.3 | -1.3 | 0.1 | 0.142372 |
| Cachexia | 1999 | 2004 | -3.5 | -6.2 | -2.3 | 0 |
| Cachexia | 2004 | 2013 | -1.1 | -1.6 | 0.8 | 0.168766 |
| Cachexia | 2013 | 2020 | -3.9 | -4.9 | -3.1 | 0 |

**References:**

1. Siegel RL, Giaquinto AN, Jemal A. Cancer statistics, 2024. CA: a cancer journal for clinicians. 2024 Jan 1;74(1).
2. Suzuki H, Asakawa A, Amitani H, Nakamura N, Inui A. Cancer cachexia—pathophysiology and management. Journal of gastroenterology. 2013 May;48:574-94.
3. Fearon K, Strasser F, Anker SD, Bosaeus I, Bruera E, Fainsinger RL, Jatoi A, Loprinzi C, MacDonald N, Mantovani G, Davis M. Definition and classification of cancer cachexia: an international consensus. The Lancet oncology. 2011 May 1;12(5):489-95.
4. Arends J, Strasser F, Gonella S, Solheim TS, Madeddu C, Ravasco P, Buonaccorso L, de van der Schueren MAE, Baldwin C, Chasen M, Ripamonti CI; ESMO Guidelines Committee. Electronic address: clinicalguidelines@esmo.org. Cancer cachexia in adult patients: ESMO Clinical Practice Guidelines☆. ESMO Open. 2021 Jun;6(3):100092. doi: 10.1016/j.esmoop.2021.100092. PMID: 34144781; PMCID: PMC8233663.
5. Gannavarapu BS, Lau SK, Carter K, Cannon NA, Gao A, Ahn C, Meyer JJ, Sher DJ, Jatoi A, Infante R, Iyengar P. Prevalence and survival impact of pretreatment cancer-associated weight loss: a tool for guiding early palliative care. Journal of oncology practice. 2018 Apr;14(4):e238-50.
6. Vagnildhaug OM, Balstad TR, Almberg SS, Brunelli C, Knudsen AK, Kaasa S, Thronæs M, Laird B, Solheim TS. A cross-sectional study examining the prevalence of cachexia and areas of unmet need in patients with cancer. Supportive Care in Cancer. 2018 Jun;26:1871-80.
7. SEER [Internet]. [cited 2025 Oct 1]. Cancer of Any Site - Cancer Stat Facts. Available from: https://seer.cancer.gov/statfacts/html/all.html
8. Centers for Disease Control and Prevention (CDC). About multiple cause of death, 1999-2020. Available at: <https://wonder.cdc.gov/mcd-icd10.html> Accessed . Accessed August 05, 2023
9. Nusrat K, Khan R, Farhan SH, Malik S, Paryani NS, Siddiq MA, Memon RS, Jawaid H, Hameed I. Disparities in the renal cancer mortality between Black and White adults in the United States, 1999-2020. J Geriatr Oncol. 2024 Jul;15(6):101797. doi: 10.1016/j.jgo.2024.101797. Epub 2024 May 23. PMID: 38789328..
10. Ingram DD, Franco SJ. 2013 NCHS Urban-Rural Classification Scheme for Counties. Vital Health Stat 2. 2014 Apr;(166):1-73. PMID: 24776070.
11. Anderson RN, Rosenberg HM. Age standardization of death rates: implementation of the year 2000 standard. Natl Vital Stat Rep. 1998 Oct 7;47(3):1-16, 20. PMID: 9796247.
12. Siegel RL, Miller KD, Fuchs HE, Jemal A. Cancer statistics, 2022. CA: a cancer journal for clinicians. 2022 Jan 1;72(1).
13. Joinpoint Regression Program, Version 4.9.0.0 - March 2021; Statistical Methodology and Applications Branch, Surveillance Research Program, National Cancer Institute. Available at: <https://surveillance.cancer.gov/joinpoint/> Accessed August 05, 2023
14. Zhong X, Zimmers TA. Sex differences in cancer cachexia. Current Osteoporosis Reports. 2020 Dec;18:646-54.
15. Permuth, J.B., Clark Daly, A., Jeong, D., Choi, J.W., Cameron, M.E., Chen, D.T., Teer, J.K., Barnett, T.E., Li, J., Powers, B.D. and Kumar, N.B., 2019. Racial and ethnic disparities in a state‐wide registry of patients with pancreatic cancer and an exploratory investigation of cancer cachexia as a contributor to observed inequities. *Cancer medicine*, *8*(6), pp.3314-3324.
16. Permuth, J.B., Clark Daly, A., Jeong, D., Choi, J.W., Cameron, M.E., Chen, D.T., Teer, J.K., Barnett, T.E., Li, J., Powers, B.D. and Kumar, N.B., 2019. Racial and ethnic disparities in a state‐wide registry of patients with pancreatic cancer and an exploratory investigation of cancer cachexia as a contributor to observed inequities. *Cancer medicine*, *8*(6), pp.3314-3324.
17. Parast L, Mathews M, Martino S, Lehrman WG, Stark D, Elliott MN. Racial/ethnic differences in emergency department utilization and experience. Journal of general internal medicine. 2022 Jan 1:1-8.
18. Patel HJ, Patel BM. TNF-α and cancer cachexia: Molecular insights and clinical implications. Life sciences. 2017 Feb 1;170:56-63.
19. Bossi P, Delrio P, Mascheroni A, Zanetti M. The spectrum of malnutrition/cachexia/sarcopenia in oncology according to different cancer types and settings: a narrative review. Nutrients. 2021 Jun 9;13(6):1980.
20. Gilmore LA, Olaechea S, Gilmore BW, Gannavarapu BS, Alvarez CM, Ahn C, Iyengar P, Infante RE. A preponderance of gastrointestinal cancer patients transition into cachexia syndrome. Journal of Cachexia, Sarcopenia and Muscle. 2022 Dec;13(6):2920-31.
21. Le‐Rademacher, J., Lopez, C., Wolfe, E., Foster, N.R., Mandrekar, S.J., Wang, X., Kumar, R., Adjei, A. and Jatoi, A., 2020. Weight loss over time and survival: a landmark analysis of 1000+ prospectively treated and monitored lung cancer patients. *Journal of Cachexia, Sarcopenia and Muscle*, *11*(6), pp.1501-1508.
22. Thongb JY, Mbengad YH, Salehd S. Gender, Racial, and Geographical Disparities in Malignant Brain Tumor Mortality in the USA. Oncology. 2024;102:703-9.
23. Guo F, Kuo YF, Shih YC, Giordano SH, Berenson AB. Trends in breast cancer mortality by stage at diagnosis among young women in the United States. Cancer. 2018 Sep 1;124(17):3500-9.
24. Gradishar WJ, Anderson BO, Blair SL, Burstein HJ, Cyr A, Elias AD, Farrar WB, Forero A, Giordano SH, Goldstein LJ, Hayes DF. Breast cancer version 3.2014. Journal of the National Comprehensive Cancer Network. 2014 Apr 1;12(4):542-90.
25. Nakajima N. Differential Diagnosis of Cachexia and Refractory Cachexia and the Impact of Appropriate Nutritional Intervention for Cachexia on Survival in Terminal Cancer Patients. Nutrients. 2021 Mar 12;13(3):915. doi: 10.3390/nu13030915. PMID: 33808957; PMCID: PMC8000003.
26. Ho SY, Guo HR, Chen HH, Peng CJ. Nutritional predictors of survival in terminally ill cancer patients. J Formos Med Assoc. 2003 Aug;102(8):544-50. PMID: 14569319.
27. Beck FK, Rosenthal TC. Prealbumin: a marker for nutritional evaluation. Am Fam Physician. 2002 Apr 15;65(8):1575-8. Erratum in: Am Fam Physician 2002 Dec 15;66(12):2208. PMID: 11989633.
28. Roeland EJ, Bohlke K, Baracos VE, Bruera E, Del Fabbro E, Dixon S, Fallon M, Herrstedt J, Lau H, Platek M, Rugo HS, Schnipper HH, Smith TJ, Tan W, Loprinzi CL. Management of Cancer Cachexia: ASCO Guideline. J Clin Oncol. 2020 Jul 20;38(21):2438-2453. doi: 10.1200/JCO.20.00611. Epub 2020 May 20. PMID: 32432946.
29. Schuppener LM, Olson K, Brooks EG. Death certification: errors and interventions. Clinical medicine & research. 2020 Mar 1;18(1):21-6.
30. Noymer A, Penner AM, Saperstein A. Cause of death affects racial classification on death certificates. PLoS One. 2011 Jan 26;6(1):e15812.
